# Bone marrow biopsy superiority over PET/CT in predicting progression‐free survival in a homogeneously‐treated cohort of diffuse large B‐cell lymphoma

**DOI:** 10.1002/cam4.1205

**Published:** 2017-09-27

**Authors:** Tzu‐Hua Chen‐Liang, Taida Martín‐Santos, Andrés Jerez, Guillermo Rodríguez‐García, Leonor Senent, Cristina Martínez‐Millán, Begoña Muiña, Mayte Orero, Anabel Teruel, Alejandro Martín, Joaquín Gómez‐Espuch, Kyra Kennedy, Carmen Benet, José María Raya, Marta Fernández‐González, Fátima de la Cruz, Marta Guinot, Carolina Villegas, Isabel Ballester, Mónica Baile, María Moya, Javier López‐Jiménez, Laura Frutos, José Luis Navarro, Jon Uña, Rosa Fernández‐López, Carolina Igua, José Contreras, Raquel Sánchez‐Vañó, María del Puig Cozar, Pilar Tamayo, Jorge Mucientes, José Javier Sánchez‐Blanco, Elena Pérez‐Ceballos, Francisco José Ortuño

**Affiliations:** ^1^ Servicio de Hematología y Oncología Médica. H.J.M. Morales Meseguer IMIB‐Arrixaca Murcia Spain; ^2^ Servicio de Hematología. H. Universitario de Canarias La Laguna Tenerife Spain; ^3^ Servicio de Hematología. H. Virgen del Rocio Sevilla Spain; ^4^ Servicio de Hematología. H. La Fe Valencia Spain; ^5^ Servicio de Hematología. H. Sta. Lucia Cartagena Spain; ^6^ Servicio de Hematología. H. R. Méndez Lorca Murcia Spain; ^7^ Servicio de Hematología. H. General Valencia Spain; ^8^ Servicio de Hematología y Oncología Médica. H. Clínico Valencia Spain; ^9^ Servicio de Hematología. H. Clínico Universitario de Salamanca/IBSAL Salamanca Spain; ^10^ Servicio de Hematología. H. Virgen de la Arrixaca Murcia Spain; ^11^ Servicio de Hematología. H. Ramon y Cajal Madrid Spain; ^12^ Servicio de Hematología. H. Arnau de Vilanova Valencia Spain; ^13^ Servicio de Medicina Nuclear. H. Virgen de la Arrixaca Murcia Spain; ^14^ Servicio de Medicina Nuclear. H. Universitario N.S. de la Candelaria Tenerife Spain; ^15^ Servicio de Medicina Nuclear. H‐ Virgen del Rocio Sevilla Spain; ^16^ Servicio de Medicina Nuclear. H. La Fe Valencia Spain; ^17^ Servicio de Medicina Nuclear. H. Sta Lucia Cartagena Murcia Spain; ^18^ Servicio de Medicina Nuclear. H. 9 de Octubre Valencia Spain; ^19^ Servicio de Medicina Nuclear. H. General‐ERESA Valencia Spain; ^20^ Servicio de Medicina Nuclear. H. Clínico Universitario de Salamanca/IBSAL Salamanca Spain; ^21^ Servicio de Medicina Nuclear. H. Puerta de Hierro Madrid Spain

**Keywords:** Bone marrow biopsy, diffuse large B‐cell lymphoma, outcomes research, PET/CT

## Abstract

Several studies have reported uneven results when evaluating the prognostic value of bone marrow biopsy (BMB) and PET/CT as part of the staging of diffuse large B‐cell lymphoma (DLBCL). The heterogeneity of the inclusion criteria and not taking into account selection and collinearity biases in the analysis models might explain part of these discrepancies. To address this issue we have carried a retrospective multicenter study including 268 DLBCL patients with a BMB and a PET/CT available at diagnosis where we estimated both the prognosis impact and the diagnostic accuracy of each technique. Only patients treated with R‐CHOP/21 as first line (*n* = 203) were included in the survival analysis. With a median follow‐up of 25 months the estimated 3‐year progression‐free survival (PFS) and overall survival (OS) were 76.3% and 82.7% respectively. In a multivariate analysis designed to avoid a collinearity bias with IPI categories, BMB‐BMI [bone marrow involvement](+) (HR: 3.6) and ECOG PS > 1 (HR: 2.9) were independently associated with a shorter PFS and three factors, age >60 years old (HR: 2.4), ECOG PS >1 (HR: 2.4), and abnormally elevated B2‐microglobulin levels (HR: 2.2) were independently associated with a shorter OS. In our DLBCL cohort, treated with a uniform first‐line chemotherapy regimen, BMI by BMB complemented performance status in predicting those patients with a higher risk for relapse or progression. In this cohort BMI by PET/CT could not independently predict a shorter PFS and/or OS.

## Introduction

In the up‐front workup of B‐cell Non‐Hodgkin lymphoma (B‐NHL), evaluating the extension of the disease is crucial because of its impact on prognosis and management. Therefore, assessing bone marrow involvement (BMI), an extranodal site, is critical to establish stage and therefore outcome 1, 2.

The impact of both 18‐F fluorodeoxyglucose (FDG) positron emission tomography with computed tomography (PET/CT) and bone marrow biopsy (BMB) in evaluating BMI in newly diagnosed B‐cell high grade lymphoma patients is at the moment a matter of intense debate 3, 4, 5. Given the lack of a “gold‐standard” to examine the most accurate test to assess BMI at diagnosis, the prognostic value of each technique emerges as a key factor to determine the correct management at baseline. Recently, several studies have reported different results when evaluating the prognostic value of BMB and PET/CT in the staging of diffuse large B‐cell lymphoma (DLBCL) 6, 7, 8, 9. The retrospective nature of the studies, the heterogeneity of the inclusion criteria, and not taking into account the collinearity/multicollinearity of variables in the multivariate regression analysis might explain part of these discrepancies.

We have extended our previously reported DLBCL multicenter series with the aim of further evaluating the impact of both techniques on progression‐free and overall survival, refining the assessment criteria, and re‐checking accuracy 10.

## Materials and Methods

### Patient populations

Two hundred and sixty‐eight consecutive patients with a diagnosis of DLBCL between August 2007 and June 2015 were retrospectively enrolled in this study from 12 tertiary centers of Spain. Patients 18 years old and older were included in the study if both BMB and PET/CT were performed simultaneously (time interval between both procedures less than or equal to 30 days) as part of the routine pre‐therapy staging for newly diagnosed DLBCL. Patients had received neither chemotherapy nor corticosteroids and no concomitant malignancy was known to be present at the time of both procedures. Pathology and PET/CT results were unknown to each other specialist.

### Bone marrow biopsy

So far, in Spain, unilateral posterior iliac crest trephine biopsy and marrow aspirate are recommended pre‐therapeutically in newly diagnosed patients with NHL according to GELTAMO guidelines.

BMBs were evaluated by an experienced hematopathologist in each hospital; results were obtained from the individual reports and were not reviewed thereafter. Data from bone marrow aspirate, flow cytometry, or molecular analysis were not used for the analysis in this work.

### PET/CT imaging and analysis

PET/CT studies were carried out using the following PET/CT devices: Gemini TF64, Gemini GXL, and Gemini TF16 (all three Gemini devices from Philips), Discovery LS (GE Healthcare), and either Biograph mCT 20 Flow, Biograph TP16 and Biograph 6 (the last three from Siemens). Procedure, quality control, and interpretation guidelines are commented in detail in our previous work 10. Briefly, the low‐dose CT components of the PET/CT were used for both co‐localization and attenuation correction of the PET emission data.

Coronal, sagittal, and transversal PET/CT projections were reconstructed by iterative methods and analyzed using the manufacturers′ software. Image interpretation was performed by qualitative (visual) analysis, considering the presence or absence of BMI using glucose activity in the liver as a reference. If present, bone marrow lesions were characterized as focal lesions or diffuse uptake exceeding that of the liver. Semiquantitative analysis was performed by means of the maximum standardized uptake value (SUVmax), normalized to body weight, as the voxel with maximum uptake in a region or volume of interest.

### Data analysis and ethics

Overall survival (OS) was defined as the time from diagnosis to death of any cause or the last follow‐up date. Progression‐free survival (PFS) was defined as the time from diagnosis to first relapse or progression, death of any cause or last follow‐up date.

OS and PFS were analyzed using the Kaplan–Meier estimate survival curves with the log‐rank test according to either BMB or PET/CT bone marrow status. Multivariate Cox proportional hazards regression models were performed including sex, B2microglobulin upper limit of normal (>2.5 mg/L), presence of bulky mass (≥10 cm), BMB and PET/CT bone marrow status, and IPI factors [age, LDH upper limit of normal (>250 UI/L), ECOG performance status (>1 score), and number of extranodal sites and Ann Arbor stage assessed by PET/CT and BMB] 11. To avoid collinearity in the Cox models, we deconstructed the IPI composite by not considering the BMI in the score of the extranodal site and Ann Arbor Stage categories. We used a *P* value threshold of <0.150 in the univariate analysis for a factor to enter the multivariate model, where *P* values less than 0.05 were considered significant.

The final diagnosis of BMI by lymphoma was achieved in cases of positive PET/CT or positive BMB 6. No guided biopsies were considered for the analysis. All performance tests were calculated as previously stated 10. Statistical analysis was done using SPSS software (IBM SPSS Statistics 21, IBM Corporation, Chicago, IL) and Epidat 3.1 (http://dxsp.sergas.es). Data were obtained by review of medical records. This study was approved by the Hospital Morales Meseguer Ethics Committee and thereafter by each of the involved hospitals.

## Results

### Patient characteristics

A total of 268 DLCBL patients were initially identified from all institutions. Performance of BMB and PET/CT was assessed in this population (see below). Main characteristics at baseline are referred in Table 1.

**Table 1:** Baseline characteristics at diagnosis

Characteristics	Global series (*N* = 268)	R‐CHOP/21 cohort^3^ (*n* = 203)
Age at diagnosis, median (range)	61 (18–85)	61 (18–85)
Female/male, *n* (%)	135 (50.4)/133 (49.6)	102 (50.2)/101 (49.8)
WBC (×10E9/L), median (range)	7 (1.3–28.2)	6.9 (1.3–20.8)
Hb (g/dL), median (range)	12.2 (7.6–16.7)	12.4 (7.7–16.7)
Platelets (×10E9/L), median (range)	250 (15–965)	245 (27–802)
B2microglobulin [ULN]^1^, *n* (%)	145 (54.1)	104 (51.2)
LDH [ULN]^2^, *n* (%)	176 (65.7)	131 (64.5)
ECOG PS, *n* (%)		
0–1	222 (82.8)	175 (86.2)
2–4	46 (17.2)	28 (13.8)
B symptoms, *n* (%)	88 (32.8)	61 (30.0)
Stage (Ann Arbor), *n* (%)		
I–II	67 (25.0)	51 (25.1)
III–IV	201 (75.0)	152 (74.9)
Bulky mass [≥10 cm], *n* (%)	72 (26.9)	51 (25.1)
Extranodal involvement (sites), *n* (%)		
0–1	215 (80.2)	168 (82.8)
≥2	53 (19.8)	35 (17.2)
Splenomegaly, *n* (%)	73 (27.2)	52 (25.6)
IPI, *n* (%)		
Low (0, 1)	74 (27.6)	60 (29.6)
Low‐intermediate (2)	82 (30.6)	60 (29.6)
High‐intermediate (3)	66 (24.6)	55 (27.1)
High (4, 5)	46 (17.2)	28 (13.8)
BMI by BMB, *n* (%)	35 (13.1)	26 (12.8)
BMI by PET/CT, *n* (%)	60 (22.4)	42 (20.7)
Diffuse	15 (5.6)	10 (4.9)
Focal	45 (16.8)	32 (15.8)
Unifocal	9 (3.4)	8 (3.9)
Multifocal	36 (13.4)	24 (11.8)
SUV max, median (p25–p75)		
Diffuse	6.7 (4.3–9.8)	6.8 (5.1–9.8)
Focal	9.8 (6.3–15.3)	10.6 (4.9–16.0)

ECOG PS, Eastern cooperative oncology group performance score; LDH, lactate dehydrogenase; ULN, upper limit of normal (^1^B2microglobulin > 2.5 mg/L and ^2^LDH>250 UI/L); IPI, international prognosis index; BMI, bone marrow involvement; BMB, bone marrow biopsy; PET/CT, positron emission tomography/computed tomography; SD, standard deviation.

^3^Patients treated with R‐CHOP/21 as first‐line therapeutic strategy.

**Figure 1:**
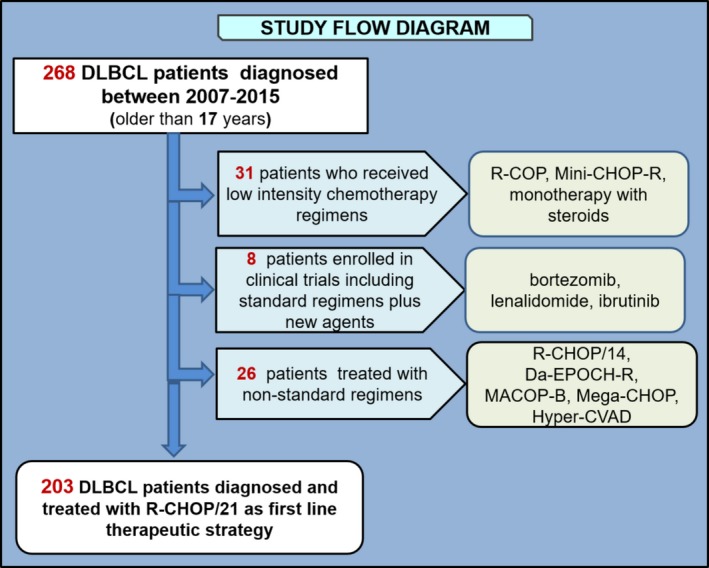
Study flow diagram. Visual representation of the exclusion criteria (left) and chemotherapy regimens (right).

For the survival analysis assessment, only patients homogeneously treated with R‐CHOP/21 (rituximab plus cyclophosphamide, doxorubicin, vincristine, and prednisone) were included. We excluded for this subanalysis: 31 patients who received low‐intensity chemotherapy regimens (R‐COP, Mini‐CHOP‐R, monotherapy with steroids) due to advanced age, comorbidities or fragility; 34 patients were also excluded because they were enrolled in clinical trials including standard regimens plus new agents (Bortezomib, Lenalidomide, Ibrutinib) or nonstandard regimens (R‐CHOP/14, Da‐EPOCH‐R, MACOP‐B, Mega‐CHOP, Hyper‐CVAD) (Fig. 1).

The remaining 203 DLBCL patients, homogeneously treated with R‐CHOP/21 showed a median age at diagnosis of 61 years old (range 18–85), with a balanced gender distribution (102 females/101 males). They received a median number of 6 cycles (range 1–8). Twenty‐eight patients (13.8%) had an IPI score of 4–5. Approximately, three‐quarters of patients (74.9%) were scored as stage III or IV according to Ann Arbor classification and 131 (64.5%) had elevated lactate dehydrogenase (LDH). The baseline characteristics of patients included in the global series and the subgroup who were treated with R‐CHOP/21 as first‐line treatment are shown in Table 1.

### Survival analysis

**Figure 2:**
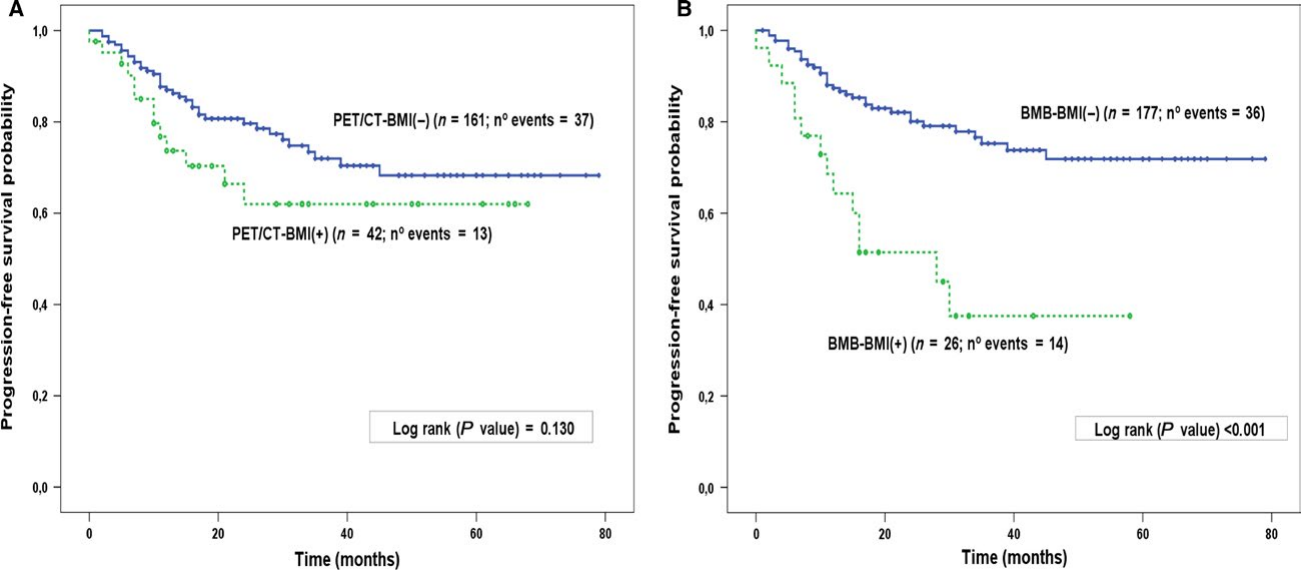
Kaplan–Meier estimates of PFS in R‐CHOP/21(*N* = 203), treated DLBCL according to BMI with PET/CT (A) or bone marrow biopsy (B). BMI, bone marrow involvement; BMB, bone marrow biopsy; PET/CT, positron emission tomography/computed tomography.

With a median follow‐up of 25 months (1–91 months) for the DLBCL patients treated with R‐CHOP/21 as first‐line therapeutic strategy, 50 patients (24.6%) progressed or relapsed and 41 (20.2%) died. The estimated 3‐year progression‐free survival (PFS) and overall survival (OS) were 76.3% and 82.7%, respectively. By univariate analysis, factors associated with a shorter PFS, with a *P* < 0.150, were: female gender, age >60 years old (more than 60 years old), ECOG PS > 1, both increased LDH and B2‐microglobulin levels, PET/CT‐BMI(+) and BMB‐BMI(+). In multivariate analysis, only two factors, BMB‐BMI(+) (HR: 3.6, 95% CI 1.7–7.6; *P* = 0.001) and ECOG PS > 1 (HR: 2.9, 95% CI 1.5–6.0; *P* = 0.003) were independently associated with a shorter PFS. By univariate analysis, predictive factors for a shorter OS, with a *P* < 0.150, included: age > 60 years old, ECOG PS > 1, increased LDH, elevated B2‐microglobulin levels and PET/CT‐BMI(+). In multivariate analysis, only three factors, age > 60 years old (HR: 2.4, 95% CI 1.2–4.8; *P* = 0.010), ECOG PS > 1 (HR: 2.4, 95% CI 1.2–5.0; *P* = 0.017) and abnormally elevated B2‐microglobulin levels (HR: 2.2, 95% CI 1.0–4.5; *P* = 0.040) were independently associated with a shorter OS (Table 2). Kaplan–Meier curves of PFS depending on BMB or PET/CT BMI are shown in Figure 2.

**Table 2:** Univariate and multivariate Cox regression analysis for PFS and OS for patients treated with R‐CHOP/21 as first‐line therapeutic strategy (*n *=* *203)

Characteristics	Progression‐free survival			Overall survival		
	Univariate	Multivariate	HR (95% CI)	Univariate	Multivariate	HR (95% CI)
Female/male	0.010	NS	–	0.569	–	–
Stage IV‐ non BMI	0.583	–	–	0.755	–	–
Age > 60	0.018	NS	–	0.004	0.010	2.4 (1.2–4.8)
ECOG PS (>1)	<0.001	0.003	2.9 (1.5–6.0)	<0.001	0.017	2.4 (1.2–5.0)
B2microglobulin [ULN]^a^	0.017	NS	–	<0.001	0.040	*2.2 (1.0–4.5)*
Extranodal‐Non BMI (>1 site)	0.370	–	–	0.268	–	–
LDH [ULN]^a^	0.124	NS	–	0.005	NS	–
Bulky mass [≥10 cm]	0.151	–	–	0.504	–	–
BMI by PET/CT	0.121	NS	–	0.018	NS	–
BMI by BMB	<0.001	0.001	3.6 (1.7–7.6)	0.326	–	–

Numbers in red: *P* < 0.05; blue: *P* ≤ 0.15. ECOG PS, Eastern cooperative oncology group performance score; LDH, lactate dehydrogenase; ULN, upper limit of normal (^1^B2microglobulin > 2.5 mg/L and ^2^LDH > 250 UI/L); BMI, bone marrow involvement; BMB, bone marrow biopsy; PET/CT, positron emission tomography/computed tomography; NS, no significance.

### Performance of PET/CT and BMB

In the global series (*N* = 268), 35 patients (13.1%) had BMI on BMB, whereas 60 (22.4%) had BMI according to PET/CT findings. Seventy‐one patients (26.5%) had BMI according to either BMB or PET/CT. Concordant BMI by means of both techniques was present in 24 (8.9%) patients. In Table 3, we specified the distribution of uptake according to BMB and PET/CT in the global series and in the R‐CHOP/21 cohort. In patients with BMI according to PET/CT, 15 (5.6%) had a diffuse pattern and 45 (16.8%) focal lesions. Among the 60 PET/CT positive patients, the median maximum standard uptake value (SUVmax) was 9.5 (p25–p75; 5.9–13.5); out of these, 45 and 15 showed a focal [median SUVmax, 9.8 (p25–p75;6.3–15.3)] and a diffuse [median SUVmax 6.7 (p25–p75; 4.3–9.8)] involvement, respectively (Table 1). Within these 60 patients, 3 had SUVmax ≤ 4 with focal uptake and 1 with a diffuse pattern.

**Table 3:** Distribution of uptake according to BMB and PET/CT

PET/CT	Global series (*N* = 268)			R‐CHOP/21 coHORT^a^ (*n* = 203)		
	BMB					
	Negative	Positive	Total	Negative	Positive	Total
Negative	197	11	208	151	10	161
Positive	36	24	60	26	16	42
Total	233	35	268	177	26	203

BMB, bone marrow biopsy; PET/CT, possitron emission tomography/computer tomography.

a Patients treated with R‐CHOP/21 as first‐line therapeutic strategy.

Among the 36 patients with BMI by PET/CT and negative BMB, 2 with diffuse and 12 with focal uptake were upstaged from stage II to stage IV, respectively, among the 11 patients with negative PET/CT and positive BMB, 5 were upstaged from stage II to stage IV.

Considering the performance by PET/CT, the sensitivity was 68.6% [95% confidence interval (CI); 51.8–85.4], specificity was 84.5% [95% CI; 79.7–89.4], NPV was 94.7% [95% CI; 91.4–98.0], accuracy 82.5% [95% CI; 77.7–87.2] and Youden Index (YI) was 0.5 [95% CI; 0.4–0.7]. Regarding BMB, the sensitivity was 40.0% [95% CI; 26.8–53.2], specificity was 94.7% [95% CI; 91.4–98.0], NPV was 84.5% [95% CI; 79.7–89.4], accuracy 82.5 [95% CI; 77.7–87.2] and YI was 0.35 [95% CI; 0.2–0.5].

## Discussion

In the setting of B‐NHL, and particularly in the DLBCL category, the use of PET/CT and/or BMB for staging purposes is presently one of the most debated issues as different strategies have been proposed according to the divergent results of a number of distinct series or meta‐analysis 3, 4, 5, 6, 7, 8, 12, 13, 14, 15, or experience‐based recommendations from experts in the field 4, 5, 16, 17, 18, 19, 20. With an endeavor to avoid susceptibility and collinearity biases in our study design, we show here the superiority of BMB over PET/CT in predicting PFS in a homogeneously‐treated cohort of 203 DLBCL patients.

Diagnostic performance has been considered an argument to abrogate the routine use of BMB in this setting due to a claimed superiority of PET/CT. However, we and others have shown before that the performance of PET/CT in terms of sensitivity and accuracy was not as high as stated in other series 10, 12, 13. To this regard, it has been proposed that this could rely in histologically unproven cases that could have been inappropriately considered true positives 12, 19, 21. Our present results regarding accuracy are very close to our previous ones and reinforce the suboptimal performance of both PET/CT and BMB. Regarding the raw numbers relating stage, 11 out of 268 DLBCL patients would have been understaged if using only PET/CT and this would have been the case for 36 patients if only BMB would have been used.

One consistent criticism regarding previous works addressing only diagnostic performance, including ours, was that independently of accuracy, PET/CT did not impact survival 3, 5, 19. However, in our view, this criticism was based on conflicting data as a number of papers have shown that lack of prognostic value 6, 7, whereas others did find it 8, 9. Interestingly, the largest series study in the setting of DLBCL so far sustains the abandon of the routine use of BMB based on the impact of PET/CT on both OS and PFS, and the unlikelihood of BMB upstaging or driving clinically significant changes 9. The inconsistency of data in the current literature can be explained by some methodological issues. A multivariable analysis is the most favored approach when assessing associations between risk factors and disease end points. However, the efficiency of multivariable models depends on the correlation architecture among potentially predictive variables. The situation in which what a regressor explains about the response is overlapped by what another regressor does is called collinearity, which ultimately leads to biased estimations 22. Adding BMI by BMB and/or PET/CT to all the component clinical indicators from the IPI in a multivariate model would inarguably suffer from this bias, as BMI is a part of the extranodal site and Ann Arbor Stage IPI clinical indicators. To our knowledge, only Khan et al. and Berthet et al. have addressed this building‐block bias by studying the impact of BMI on prognosis exclusively in stage IV patients and separately assessing each IPI factor prognostic value, respectively 6, 8. We chose to “deconstruct” the IPI composite by not considering the BMI in the extranodal site and Ann Arbor Stage categories. This is, in our view, the most accurate procedure for a correct assessment of the prognostic weight of each BMI detection technique.

We recognize that our current results have modified our view regarding the up‐front workup in the DLBCL setting. Though in our previous work, and in line with others 12, 17, we suggested that a positive PET/CT could abrogate the need for a BMB, according to our present results, we now believe that BMB should be performed in all DLBCL patients at baseline. In the absence of a feasible gold standard for a methodologically indisputable assessment of the sensibility and specificity of both techniques, we now agree with Adams, et al. when stating that the lack of prognostic implications of bone marrow involvement detected by FDG‐PET/CT strongly suggests there is a considerable proportion of false‐positive FDG‐PET/CT cases in patients with DLBCL 23. In addition, it could seem contradictory that BM involvement based on BMB had prognostic impact on PFS but not on OS. However, in our view, fewer end‐point events and the inclusion of all causes of death could account for this apparent contradiction.

We acknowledge that our study design (multicenter series and retrospective nature) confers both strength and weakness to our study. We have not discriminated between germinal‐like and activated subtypes, an issue than sooner or later should be addressed in this setting. Nevertheless, the coherence of the present data with our previous work and others′ in terms of performance suggests that our results reflect “real life” clinical practice. In addition, it could be argued a relative heterogeneity regarding PET/CT procedures, particularly the lack of a unique strict protocol. In this regard, it is of note that all the NM units involved in this study are carrying out PET/CT lymphoma staging since, at least, 2006 and that analysis has been performed in all cases by experienced senior NM specialists following the European Association of Nuclear Medicine recommendations for visual and semiquantitative analysis of tumor PET imaging as we stated before. In this regard, it must be underlined that there are still no guidelines or agreement for the up‐front baseline studies of DLBCL, as stated by the uneven recommendations stated in the Lugano meeting in 2015 16, 17, and the updated NCCN guidelines.

In summary, in our DLBCL cohort, treated with a uniform first‐line chemotherapy regimen, BMI by BMB complemented IPI in predicting those patients with a higher risk for relapse or progression, while IPI defined a subset of patients with a worse survival. In this cohort, BMI by PET/CT could not independently predict for a shorter PFS and/or OS. At the present moment, both techniques seem to offer different and complementary clinical information.

## Conflict of Interest

None declared.
